# A Path to Sleep Is through the Eye^1^,^2^,^3^

**DOI:** 10.1523/ENEURO.0069-14.2015

**Published:** 2015-03-26

**Authors:** Lawrence P. Morin

**Affiliations:** Department of Psychiatry and Graduate Program in Neuroscience, Stony Brook Medicine, Stony Brook University, Stony Brook, New York 11794

**Keywords:** circadian, masking, melanin-concentrating hormone, melanopsin, orexin, sleep

## Abstract

Light has long been known to modulate sleep, but recent discoveries support its use as an effective nocturnal stimulus for eliciting sleep in certain rodents. “Photosomnolence” is mediated by classical and ganglion cell photoreceptors and occurs despite the ongoing high levels of locomotion at the time of stimulus onset. Brief photic stimuli trigger rapid locomotor suppression, sleep, and a large drop in core body temperature (Tc; Phase 1), followed by a relatively fixed duration interval of sleep (Phase 2) and recovery (Phase 3) to pre-sleep activity levels. Additional light can lengthen Phase 2. Potential retinal pathways through which the sleep system might be light-activated are described and the potential roles of orexin (hypocretin) and melanin-concentrating hormone are discussed. The visual input route is a practical avenue to follow in pursuit of the neural circuitry and mechanisms governing sleep and arousal in small nocturnal mammals and the organizational principles may be similar in diurnal humans. Photosomnolence studies are likely to be particularly advantageous because the timing of sleep is largely under experimenter control. Sleep can now be effectively studied using uncomplicated, nonintrusive methods with behavior evaluation software tools; surgery for EEG electrode placement is avoidable. The research protocol for light-induced sleep is easily implemented and useful for assessing the effects of experimental manipulations on the sleep induction pathway. Moreover, the experimental designs and associated results benefit from a substantial amount of existing neuroanatomical and pharmacological literature that provides a solid framework guiding the conduct and interpretation of future investigations.

## Abstract (precis)

Sleep is expressed as a circadian rhythm and the two phenomena exist in a poorly understood relationship. Light affects each, simultaneously influencing rhythm phase and rapidly inducing sleep.

## Significance Statement

Sleep is expressed as a circadian rhythm and the two phenomena exist in a poorly understood relationship. Light affects each, simultaneously influencing rhythm phase and rapidly inducing sleep. Here, multiple effects of light on sleep are discussed, as are the possible anatomical pathways by which photic input might reach brain-stem sleep-regulating nuclei. Emphasis is placed on a simple procedure by which a brief light stimulus rapidly induces sleep. Studies of the photic input pathway through which light elicits sleep, combined with modern software and hardware tools enabling studies of behavioral sleep without EEG and neurosurgery, are likely to provide a very productive avenue for probing the mechanisms of sleep onset and regulation.

## Introduction

Complex physiological and behavioral activities are commonly influenced by simple environmental stimuli. Such is the case for the generation and regulation of sleep. In nocturnal rodents, sleep is expressed primarily during the hours of daylight, with abundant locomotor activity occurring during the night. Two organizing principles governing sleep are confirmed by this information. One is regulation of sleep timing through photoperiod-induced entrainment of the baseline sleep/wake circadian rhythm. The other is the presence of temporal incompatibility between sleep and locomotion such that an active animal does not sleep and, if it sleeps, it is not active. Active animals are not sleeping animals and, by implication, sleep should be difficult to induce in active animals. Unexpectedly, the opposite is true: exposing highly active mice or hamsters to nocturnal light causes their activity to cease in association with sleep induction.

Recent research suggests that the use of photic stimulation in sleep studies may elucidate a neural circuit originating in ocular photoreceptors and projecting to the complex neural system generating and regulating sleep. A more thorough understanding of this circuit can form the basis of a research strategy designed to ultimately describe the structure and operation of the sleep-regulatory system.

This presentation (1) provides a status report regarding light-induced sleep; (2) advocates on behalf of simple sleep research methods that emphasize behavioral analysis; (3) reinforces the view that visual projections provide a fairly direct path from photoreceptors to brain regions and neural circuitry responsible for sleep generation and regulation; and (4) suggests that simple studies of light-induced sleep offer a unique opportunity to study the mechanisms underlying sleep. Nocturnal light exposure creates a convenient avenue through which entry into and emergence from the sleep state can be stimulated, explored, and understood.

## Photic Modification of Sleep in Nocturnal Rodents

Light has been known to modulate sleep for many years, with many of its effects documented in works from the Borbély lab and summarized in his review, “Effects of light on sleep and activity rhythms.” In it, Borbély (1978) describes four lighting effects. Most obvious is the influence of the daily photoperiod on entrainment of the central circadian clock in the suprachiasmatic nucleus (SCN) and the consequent timing of the sleep/wake rhythm (see also Mistlberger, 2005).

Borbély also described two additional lighting effects in rats exposed to alternating light and dark (LD) intervals multiple times per 24 h day (ultradian LD photoperiods). Rapid eye movement (REM) sleep can be triggered simply by turning off the light (Lisk and Sawyer, 1966; Borbély et al., 1975; Deboer et al., 2007), as occurs repeatedly when animals are housed under a 15 min lights on/15 min lights off photoperiod (for review, see Baracchi et al., 2008).

The experimental design favored by Borbély showed that certain effects of light were more likely to occur during one particular phase of the animals’ rest/activity cycle. He noted, for example, that when the 15 min of light during an ultradian LD photoperiod coincided with the nocturnal activity phase, rats became less active and showed a rapid increase in non-rapid eye movement (nREM) sleep. Light exposure during the inactive phase tended to have little to no effect on the amount of sleep. He also noted that this effect was irradiance-dependent (Borbély et al., 1975; Borbély, 1978). The basic observation that nocturnal light augments sleep while suppressing motor activity is a third effect of light and the primary concern of this presentation. Several studies employing mice and longer light exposures have confirmed Borbély’s basic observations that sleep and inactivity are induced by light exposure during the nocturnal activity phase (Altimus et al., 2008; Lupi et al., 2008; Tsai et al., 2009; van Oosterhout et al., 2012a).

In addition to the foregoing, there is a fourth lighting-related effect. Exposing sleeping mice to darkness during the normal daylight hours of the daily photoperiod has been reported to increase behavioral arousal (Altimus et al., 2008; Tsai et al., 2009). Such results support a suggestion from Borbély’s data (Borbély, 1975) that rats engage in more locomotion (and less nREM sleep) in the dark portion of ultradian LD photoperiods even during the typical subjective daytime sleeping hours.

The Borbély (1978) review discussed many topics related to photic regulation of sleep. It was deficient only with respect to knowledge about intrinsically photoreceptive retinal ganglion cells (ipRGCs) and the fact that very brief nocturnal photic stimuli simultaneously inhibited locomotion while augmenting sleep. Initial awareness of the former occurred about 20 years after Borbély’s review (Provencio et al., 1998, 2000, 2002) and almost 30 years for the latter (Vidal and Morin, 2007). It is now known that visual system regulation of activity and sleep involves contributions from rod, cone, and ipRGC photoreceptors. Nocturnal photic information triggers an abrupt transition from the awake, active state to the state of behavioral quiescence and sleep.

In addition to the emphasis of light effects on sleep, Borbély was careful to describe the “dual action of light” consisting of induction of behavioral state and alteration of circadian rhythm phase. Despite the prescience of the Borbély review, it has been cited only 90 times, although it is one of the few discussions to directly address the relationship between light, sleep, and locomotion/activity. In addition, Borbély is one of the few investigators to question and evaluate the interdependence of multiple rhythmic variables (sleep, feeding, drinking, body temperature), as well as their modulation by light (Borbély and Huston, 1974; Borbély, 1978).

It should be noted that generalizations about the effects of light on sleep or arousal that are deduced from studies of nocturnal species may not apply to diurnal animals (for discussion, see Shuboni et al., 2012; Langel et al., 2014). In humans, for example, nocturnal light enhances alertness (for review, see Cajochen, 2007; Hubbard et al., 2013), contrary to the effects observed in small nocturnal laboratory rodents.

## Negative Masking in Circadian Rhythm Research

Nocturnal light exposure has a well-known suppressive effect on locomotion. This phenomenon is known as “negative masking” or simply “masking” (Mrosovsky, 1999). For a variety of reasons previously discussed (Morin, 2013b), this effect of light on activity is here referred to as “locomotor suppression.” Such suppression has been most commonly studied using an efficient procedure developed by Mrosovsky (1994) in which an animal with access to a running wheel is exposed to a 1 h light pulse early in the night. The extent to which light exposure changes the nocturnal activity is calculated relative to the level of activity during the same 1 h interval of the previous night when there was no light. The locomotor suppressive effect of light is one of a variety of non-image-forming responses of the visual system and involves light detection by both classical (rod/cone) and ganglion cell photoreceptors. The combination of rod/cone and ipRGC photoreception mediates locomotor suppression as well as other non-image-forming visual responses, including circadian rhythm phase shifts, pupillary light reflex, and melatonin suppression (Redlin, 2001; Hattar et al., 2003; Panda et al., 2003; Morin and Studholme, 2011; Morin, 2013b).

Suppressive effects of light have been considered to be “acute” or “direct” effects of light (for discussion, see Mrosovsky, 1994, 1999; Shuboni et al., 2012; Hubbard et al., 2013; Morin, 2013b; Langel et al., 2014). That is, locomotor suppression has traditionally been thought to be the result of motor inhibition caused by the ambient illumination. Although the study of light-induced locomotor suppression evolved out of circadian rhythm research concerned with the process of photo-entrainment, the phenomenon has usually been considered to be unrelated to circadian oscillator function and often an impediment to its proper evaluation (Mrosovsky, 1994; Spoelstra et al., 2004). Locomotor suppression in nocturnal animals has been proposed as a means of obtaining light-induced behavioral change that complements the effects of light on circadian rhythm phase (Mrosovsky, 1999; Langel et al., 2014). Although this general perspective has been widely held for many years, no data directly support it. In fact, similarities between photic regulation of circadian rhythm phase, locomotor suppression, and pineal melatonin synthesis are sufficient to justify the presumption that a common sensory input pathway underlies all three responses (Morin, 2013b).

## A Tale of Misdirection: Negative Masking Involves Photosomnolence

The term negative masking does not facilitate our understanding of light-induced behavioral change. It implies that something is covered or cloaked without providing any specifics regarding exactly how it occurs, what has been altered, or even what is being measured. Moreover, although the term most frequently refers to an inhibitory or negative effect of light, there are other proposed varieties, with the light-induced increase in activity (“positive” masking) being the most common (Mrosovsky, 1999)).

A more serious difficulty has arisen because studies designed to evaluate the acute, supposedly inhibitory, effect of light have failed to recognize that when one behavior in a particular repertoire is suppressed, there is an obligatory simultaneous, compensatory increase in at least one alternate behavior. Mrosovsky's studies (Mrosovsky et al., 1999; Redlin and Mrosovsky, 1999b; Mrosovsky et al., 2001; Mrosovsky and Hattar, 2003; Mrosovsky and Thompson, 2008; Thompson et al., 2008) focused on wheel running and did not experimentally consider the following question: If a nocturnal animal is exposed to an hour of light early in its active phase and its locomotion is reduced to zero, what behavior is correspondingly elevated to fill the void? This question clearly implies that light-induced locomotor suppression is not necessarily the equivalent of simple behavioral or physiological inhibition. Instead, when mice or hamsters are exposed to nocturnal light, locomotor suppression is simultaneously accompanied by a compensatory increase in sleep. “Photosomnolence” is light-induced sleep (Morin and Studholme, 2009; Studholme et al., 2013). Most importantly, the existence of photosomnolence is not consistent with the historical view that negative masking represents the loss of behavior. To the contrary, photosomnolence is a consequence of change in behavioral state, with light exposure causing an abrupt switch from awake and highly active to behaviorally quiescent and asleep.

## Locomotor Suppression as a Behavioral Proxy for Sleep

Evidence of sleep behavior in masking studies was initially obtained from video recordings of mice and hamsters housed under infrared illumination and exposed to millisecond light flashes during their nocturnal activity phase (Morin and Studholme, 2009 and unpublished hamster data cited in Morin and Studholme, 2011). More recently, quantitative video-based sleep assessment [a validated alternative to the EEG (Movie 1) (Pack et al., 2007; Fisher et al., 2012)] has confirmed sleep behavior in response to brief light stimulation (Morin and Studholme, 2011). The video and behavioral evidence for light-induced sleep has been directly confirmed by conventional EEG evaluation (Altimus et al., 2008; Lupi et al., 2008; Muindi et al., 2013; Studholme et al., 2013). In addition, both light-induced locomotor suppression and EEG-determined sleep are similarly sensitive to stimulus irradiance (Mrosovsky et al., 1999; Redlin and Mrosovsky, 1999b; Mrosovsky et al., 2000; Muindi et al., 2014). The behavioral data do not correlate perfectly with EEG measures, in part because there are brief intervals immediately prior and subsequent to the period of sleep during which animals are awake, but not detectably active.

Movie 1. This video shows a hamster photosomnolence response (10 flash stimulus, each 2 ms long, delivered at equal intervals over 5 min) filmed under infrared illumination. Photosomnolence of animals with access to running wheels is measured as the interval between running offset and resumption of running. The video has been edited purely for purposes of limiting its length. To this end, some of the light flashes and many minutes of video showing a sleeping hamster have been omitted. A similar video of a mouse is provided as supplementary material to Morin and Studholme (2009). Both videos are representative, although the prevalence of post-photosomnolence stretching is not known or whether it is specific to the hamster. 10.1523/ENEURO.0069-14.2015.video.1

## Multiple Effects of Light on the Photosomnolence Pattern

A study by Redlin and Mrosovsky (1999b) contains a result not easily explained if nocturnal locomotion is directly inhibited by ambient light, as conceptualized from the historical perspective of the locomotor suppression phenomenon. Their result shows that locomotion remains suppressed for a prolonged interval after termination of the light stimulus and the return to darkness (i.e., locomotion does not recover instantly when the light is turned off). Redlin and Mrosovsky (1999b) refer to this effect as “post-pulse masking” (see their Figs. 4, 6) and it is important to the understanding of photosomnolence because it was the first indication that the locomotor response to light is more complicated than simple behavioral inhibition.

**Figure 1.**
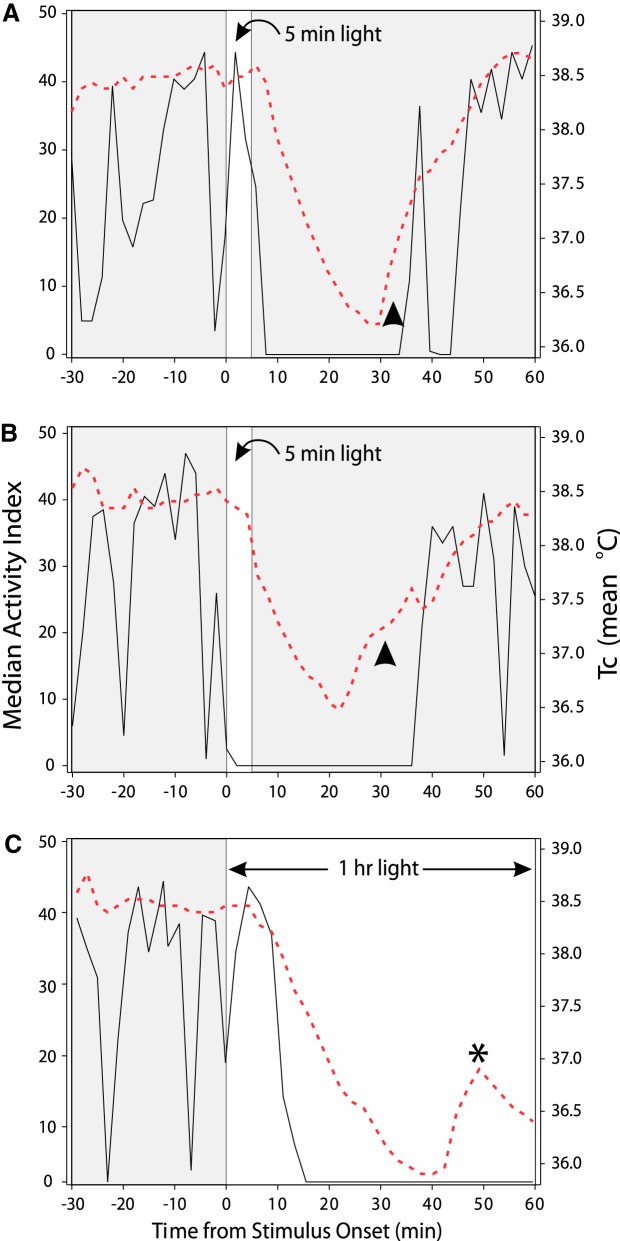
Simultaneously recorded patterns of wheel running (RPM; black solid lines) and core body temperature (red broken lines) in three individual mice housed under LD12:12 and, during the night the data were collected, exposed at ZT13 (time 0 is stimulus onset) to a 5 min light pulse (***A***, ***B***, narrow white area) and a 1 h light pulse (***C***, broad white area). The arrowheads in ***A*** and ***B*** indicate portions of the records during which Tc has risen in advance of recovered locomotion. The asterisk in ***C*** identifies a rise in Tc and an aborted return to normal. Despite the increase in Tc, the simultaneously recorded wheel running does not correspondingly increase from its level of complete suppression during light exposure. After Figure 3 in Studholme et al. (2013).

Experimental application of nocturnal photic stimuli considerably shorter than those favored by Mrosovsky has provided evidence that the typical locomotor pattern has three distinct phases (Fig. 1). Each phase has its own particular significant features that require further experimental attention. In Phase 1, the Induction phase, the initial light exposure occurs and behavioral state changes from a high arousal/activity level to behavioral quiescence and sleep. Phase two is the Sleep interval. Phase three is the interval of Recovery from sleep and the return to the pre-light level of activity.

Exposure to nocturnal light triggers Phase 1, which is characterized by a rapid loss of locomotion and transition to sleep. In Phase 1, the average level of activity is reduced over several minutes from an elevated state to zero activity and sleep. The average mouse actually increases its activity for about 60 s after the stimulus onset, with the typical locomotor decline occurring thereafter (Morin and Studholme, 2011). In other words, animals do not instantly go to sleep, falling out of their running wheels when suddenly exposed to nocturnal light (Morin and Studholme, 2009).

**Figure 2.**
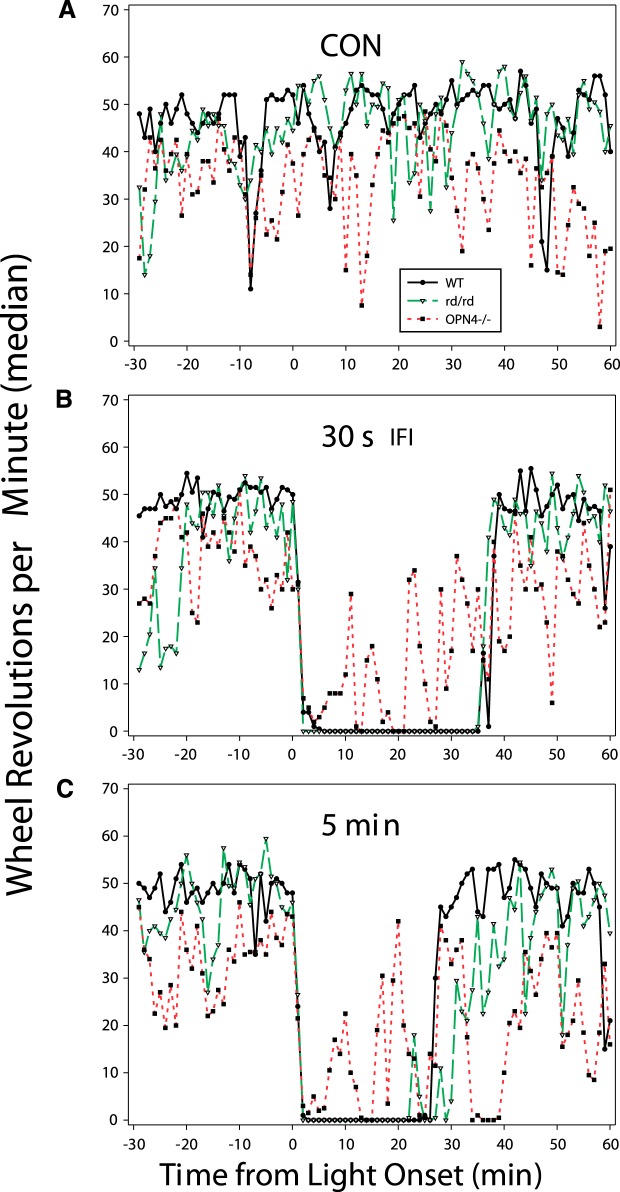
Wheel running rate by non-photostimulated mice (CON; ***A***); mice that received 10 light flashes, 2 ms each, with 30 s interflash intervals (IFI; ***B***); and mice that received a single 5 min light pulse (***C***). Photic stimulation began at time 0. The filled squares/red dotted line indicates the behavior of mice lacking melanopsin (*OPN4−/−*). The *rd/rd* mice (open triangles/green dashed line) lack rods or cones. WT, Wild-type controls (filled circles/black solid line). In ***B*** and ***C***, the *OPN4−/−* mice show a rapid locomotor suppression effect that is soon followed by erratic levels of locomotion, rather than the typical complete suppression seen in WT and *rd/rd* mice. After Figure 3 in Morin and Studholme (2011).

Phase 1 occurs in mice lacking ipRGCs or classical rod/cone photoreceptors (Fig. 2; Morin and Studholme, 2011). However, the emergent locomotor suppression/photosomnolence pattern of the average mouse lacking ipRGCs is substantially different from that of wild-type or rodless/coneless mice. On the one hand, mice lacking ipRGCs exhibit robust short-term suppression during the initial 5 min after light onset. On the other hand, absence of ipRGCs prevents the typical, prolonged light-induced, locomotor suppression. The locomotion level remains erratically suppressed for the expected duration of Phase 2 (Fig. 2B,C). These observations suggest three things: (1) Phase 1 can be triggered by either ipRGC or classical photoreceptors; (2) the duration of Phase 2 is normal if only the classical photoreceptors are absent and is more or less normal if only the ipRGCs are absent; and (3) light is a much less effective locomotion suppressor (or sleep inducer) in mice lacking only ipRGCs. The last point may indicate that differing mechanisms control the different portions of the locomotor suppression pattern (onset, duration, recovery) and the ability of the sleep induction system to be activated by light. The photosomnolence data, as indicated by behavioral sleep indices (Morin and Studholme, 2011), are consistent with additional information showing that ipRGCs are not necessary for the initiation of normal light-induced sleep as estimated from wheel running results (Mrosovsky and Hattar, 2003) or measured by EEG (Altimus et al., 2008; Lupi et al., 2008; Tsai et al., 2009; Muindi et al., 2013), but are necessary for the normal maintenance of such. In addition to the foregoing, it should be noted that only about two-thirds of mice lacking melanopsin fail to show photosomnolence; responses by the remaining third are seemingly normal (Morin and Studholme, 2011).

The interval of mouse photosomnolence (Phase 2) is about 30 min (Morin et al., 2010). It is elicited by stimuli ranging from 30 to 600 s. Briefer (≤3 s) stimuli do not effectively suppress locomotion and, by inference, are not expected to elicit sleep. Stimuli that are roughly 1200 s or longer induce an interval of robust locomotor suppression that persists while the stimulus is present, plus many minutes beyond, with the interval duration related to the length of the stimulus. Thus, exposure to light has two distinct effects on photosomnolence and locomotor suppression. The first is a suppression response of relatively fixed duration that is triggered by brief light exposure: once initiated by light, Phase 2 proceeds to completion in the absence of additional light. The second effect of light is one of additivity: once triggered, Phase 2 can be prolonged by exposure to additional light.

The duration of Phase 2 may vary with species, being about 30 min in mice (Morin and Studholme, 2009; Morin et al., 2010) and 40 min in hamsters (unpublished data cited by Studholme et al., 2013). Phase 2 ends with the beginning of Phase 3 (Recovery). Individual animals appear to wake abruptly, then move around the cage, eventually accessing the wheel and running. The length of Phase 2 can vastly exceed the stimulus duration, especially if the photic stimulus lasts only milliseconds (Vidal and Morin, 2007; Morin and Studholme, 2009; Morin and Studholme, 2014a). The average interval of mouse locomotor suppression is six times longer than a typical 5 min triggering light pulse. The ability of additional light to prolong photosomnolence automatically delays recovery. Indeed, there may be no mechanistic difference between Phase 3 and the termination of Phase 2.

## Thermoregulation during Photosomnolence

One obvious feature shown in Figure 1 is the interval of locomotor suppression triggered by light. Wheel running and general activity in the home cage are affected equally, with each rapidly falling to zero (Morin and Studholme, 2011). The decline and subsequent absence of locomotion is associated with a marked change in core body temperature (Tc; Studholme et al., 2013). This change is consistent with earlier observations (Borbély and Huston, 1974) of rats housed under ultradian LD photoperiods. The decline in Tc is of importance because of the widely held view that lower Tc facilitates sleep (Murphy and Campbell, 1997; McGinty and Szymusiak, 2003; for discussion, see Studholme et al., 2013).

On the average, Tc begins to drop several minutes after the abrupt decline in locomotion and reaches its minimum about 30 − 35 min after light stimulus onset. This contrasts with the change in wheel running, which often declines to zero in only 3 − 5 min. The magnitude of the light-induced temperature decrease varies with circadian time. Average maximal change is −1.7° in response to light exposure at circadian time 1500 (CT15) and the smallest average change (∼−0.3°) occurs during the interval of CT3−CT9 (Studholme et al., 2013). Dark exposure during the daytime has been reported to increase rat Tc, while light during the nighttime causes a Tc decrease without an associated drop in activity (Hasegawa et al., 2000). The onset of laboratory daylight is associated with an abrupt, large drop in rat brain temperature (Yoshida et al., 2001).

It is commonly suggested that Tc is causally related to the level of locomotion (Weinert and Waterhouse, 1998; Weinert and Waterhouse, 2007), but this does not seem to be the case with some species of small rodents, including the C57BL/6J laboratory mouse (Gebczynski and Taylor, 2004; Studholme et al., 2013). Five situations have been identified in which mouse Tc changes in a manner that does not reflect the level of simultaneously recorded locomotor activity. Perhaps the most obvious of these is the spontaneous Tc recovery that occurs about 5 − 10 min prior to the resumption of light-suppressed locomotion (Fig. 1A,B, arrowheads). Spontaneous Tc recovery is even more obvious if the triggering light stimulus is long (e.g., 1 h). As mentioned above, long light exposure prolongs locomotor suppression (Morin et al., 2010; Morin and Studholme, 2014a). It also delays the return of Tc to normal. However, within the temperature record, there is a transient Tc rise with no corresponding locomotion increase (Fig. 1C, asterisk). The fact that Tc then drops a second time during the longer light exposure suggests that the sleeping mice show a functional response. The alternative, that the mice momentarily awaken and open eyes before resuming sleep, has not been excluded.

It is likely that neither the abrupt decline in activity nor the sleep onset is directly responsible for the Tc drop. However, it is not at all clear whether the change in Tc contributes to the process of sleep induction. Tc and sleep are regulated by overlapping brain regions with many individual preoptic neurons contributing to both thermoregulation and sleep (for review, see Szymusiak and McGinty, 2008). Studies involving preoptic heating or cooling suggest there is a direct effect of hypothalamic temperature on sleep. Within limits, as preoptic temperature increases, sleep time increases. Preoptic temperature increases activate heat loss mechanisms, with the converse occurring in response to decreases (Sakaguchi et al., 1979; McGinty et al., 1994; Nakamura, 2011). A drop in Tc may indicate a change in hypothalamic physiology necessary for sleep, but not causal of it (see also Krauchi and Deboer, 2010). The ability to block photosomnolence by exogenously clamping Tc or hypothalamic temperature at baseline levels has not been tested.

The light-induced lower Tc is likely the consequence of a drop in the thermoregulatory set point for hypothalamic temperature. Such a drop would cause cutaneous vasodilation and body heat loss to accommodate the lower set point. The observed light-induced drop in Tc is likely a sequel to autonomically-driven (and presumably rapid) vasodilation initiated by cells in the medial preoptic region (Nakamura, 2011; Tanaka et al., 2011). Such vasodilation would be expected to elicit a short latency (≤1 min; Tanaka et al., 1986), rapid rise of skin temperature, precipitating a drop in Tc resulting from transcutaneous heat loss. Light could exert its effects directly through synaptic influences on neural activity in the circuitry controlling vasodilation or indirectly by elevating hypothalamic temperature above the existing set point, which would also cause compensatory heat loss. The temporal and causal relationships between light-induced sleep, rate of peripheral heat loss, and hypothalamic temperature change await further study.

## An Eye to Sleep: Speculative Neuroanatomy of Photosomnolence

**Figure 3.**
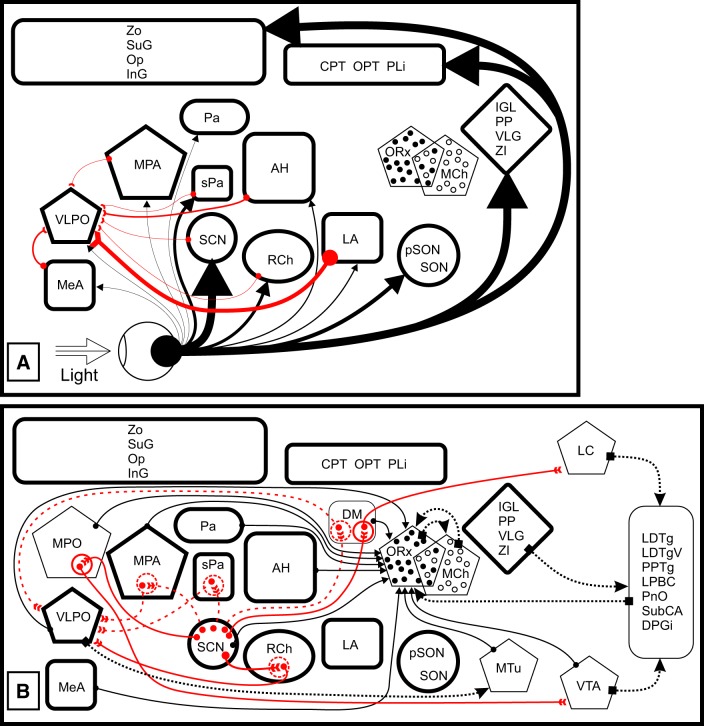
***A***, Retinal projections, retinorecipient nuclei, and second order afferents to the VLPO. Black pathways, First-order retinal projections to basal forebrain, thalamic, and visual midbrain nuclei (thick black outlines in both ***A*** and ***B***). Red pathways, Second-order projections afferent to the VLPO from retinorecipient nuclei. Projection density roughly corresponds to arrow line thickness. ***B***, Connections between forebrain nuclei that may be providing second- or third-order retinal input to the sleep regulatory system. Solid red pathways, Virally traced projections from the SCN with at least one known synapse (red circles). Broken red pathways, Possible routes from the SCN to the VLPO involving a probable, but not identified, synapse (large broken red circles). Solid black pathways, Projections to ORx cells (after Yoshida et al., 2006; there is no similar information for MCH cell innervation). Dotted black pathways, Other projections to and from sleep-regulatory nuclei. Note: All nuclei identified in the figure are innervated by both ORx and MCH cells (see Table 1). See Table 2 for anatomical abbreviations and the text for references. These schematics are not intended to exclude any other possible arrangement of connections between the visual and sleep systems.

Photosomnolence involves light detection by both classical photoreceptors and ipRGCs, with the latter funneling information from all photoreceptor types to the brain (Hattar et al., 2006; Göz et al., 2008; Güler et al., 2008; Hatori et al., 2008; Tsai et al., 2009; Morin and Studholme, 2011; Muindi et al., 2013). Retinal projections (Fig. 3A) from all types of ganglion cells are present in about 46 different mouse brain regions, including 12 in the hypothalamus (Morin and Studholme, 2014b). Many of the retinorecipient nuclei, especially those in the subcortical visual shell of the midbrain and thalamus, are reciprocally interconnected (Morin and Blanchard, 1998). In addition, there is a strong relationship between the intergeniculate leaflet (IGL) and SCN, not only because of their robust connection through the geniculohypothalamic tract, but because the two nuclei project to nearly all the same hypothalamic locations (Morin, 2013a). Except for the olivary pretectal nucleus, there are no direct retinal projections to brain stem regions associated with sleep regulation. Therefore, it is most likely that the critical input pathway for photosomnolence involves a relay in one or more of the hypothalamic nuclei to the sleep regulatory system, with the SCN being the prime candidate.

### Lesions of Retinorecipient Nuclei and Light-Induced Locomotor Suppression

From a functional perspective, virtually none of the regions thought to be elements of the sleep system have been evaluated for their contribution to light-induced sleep. Theoretically, a lesion that destroys an essential part of the circuitry through which photosomnolence is elicited could prevent both light-induced locomotor suppression and the associated sleep. For example, genetic elimination of all known photoreception prevents light-induced locomotor suppression and sleep (Hattar et al., 2003; Panda et al., 2003; Altimus et al., 2008). Similarly, the brain lesion approach has been used to test function of several parts of the visual system, including the visual cortex, pretectum, IGL, dorsal lateral geniculate, and SCN (Redlin et al., 1999; Edelstein and Mrosovsky, 2001; Redlin et al., 2003). In contrast to the effect of photoreceptor destruction, damage to the listed brain areas generally has no effect or actually increases locomotion. The exception is the SCN, where complete lesions block light-induced locomotor suppression (Li et al., 2005; but see Redlin and Mrosovsky, 1999a, who concluded that SCN absence does not block such suppression; see also Morin, 2013b for additional references and discussion of the possibility that the latter lesions may not have been complete). Although the anatomical emphasis is on the SCN, retinorecipient peri-SCN areas cannot be excluded as contributors to the regulation of photosomnolence (Morin, 2013b).

Whether or not the SCN serves as a synaptic relay for retinal input to the sleep regulatory system, it is involved in the expression of photosomnolence. Redlin and Mrosovsky (1999b) found, using conditions contrived to facilitate activity at all times of day, that a circadian clock regulates the magnitude of light-induced hamster locomotor suppression. Similarly, the light-induced decline in mouse Tc is strongly modulated by the circadian clock (Studholme et al., 2013), and the two effects in the different species are maximal at approximately the same circadian time. In the absence of functional molecular clockworks, light continues to induce locomotor suppression (Van der Horst et al., 1999; Mrosovsky, 2001), indicating that the circadian clock *per se* is not necessary for the locomotor response to light; sleep has not been examined.

#### First-Order (Direct) Retinal Projections

The direct retinal projection to the ventrolateral preoptic area (VLPO; Fig. 3A), a location from which sleep is thought to be activated (Sherin et al., 1996; Lu et al., 2000; McGinty and Szymusiak, 2001), is the most obvious candidate for photic sleep induction. This first-order input route is rendered less likely as a contributor to photosomnolence by virtue of the fact that detectable VLPO afferents from mouse retina are very sparse (Hattar et al., 2006; Morin and Studholme, 2014b), although they are less so in the hamster (Morin and Blanchard, 1999). Nocturnal light exposure induces c-fos RNA synthesis in mouse VLPO neurons (Lupi et al., 2008), but it is presently impossible to know whether this is a result of direct or indirect photic input.

#### Second-Order Projections to VLPO

Given the very dense retinal innervation throughout the SCN (Muscat et al., 2003; Hattar et al., 2006; Morin et al., 2006; Morin and Studholme, 2014b) and its widespread projections within the diencephalon (Watts and Swanson, 1987; Watts et al., 1987; Kalsbeek et al., 1993; Morin et al., 1994; Morin and Blanchard, 1999; Morin, 2013a), the SCN is a prime candidate for relaying second-order photic information to the sleep system (Fig. 3A). The SCN and nearby retinorecipient regions, particularly the retrochiasmatic area (RCh) and subparaventricular zone (sPa), send efferents to much of the medial hypothalamus, including the VLPO (Morin et al., 1994; Kriegsfeld et al., 2004). As with the direct retinal projection to the VLPO, the SCN projection might mediate photosomnolence [it provides both excitatory and inhibitory input (Sun et al., 2000, 2001)], but it is also sparse and, based on size alone, may contribute little to the light-induced response (Novak and Nunez, 2000; Chou et al., 2002). Similar issues pertain to projections from other retinorecipient peri-SCN hypothalamic structures (for review, see Morin, 2013a).

#### Third-Order Connections

Third-order photic inputs to the VLPO (Fig. 3B) may be more likely contributors to sleep induction and locomotor suppression. Nuclei innervated by the SCN and which project heavily to the VLPO include the medial preoptic area (MPA), dorsomedial hypothalamic nucleus (DM), RCh, sPa, and perifornical hypothalamus (Deurveilher et al., 2002; Deurveilher and Semba, 2003, 2005).

Additionally, transneuronal tract-tracing procedures have demonstrated at least two distinct pathways from the SCN to other nuclei of the sleep system. In one (Fig. 3B), the SCN projects to the locus coeruleus, where it may modulate arousal (Aston-Jones et al., 1995; Luppi et al., 1995; Aston-Jones et al., 2001). The second such route reaches the ventral tegmental area from the SCN (Fallon and Loughlin, 1995; Geisler and Zahm, 2005; Luo and Aston-Jones, 2009; Tsujino and Sakurai, 2009). The effects of neurotoxic lesions targeting cell bodies suggest that the DM and the MPA are relay nuclei in these circuits.

#### Orexin Pathways

The orexin (ORx; or hypocretin) cells in the lateral hypothalamus are direct targets of the SCN and constituents of the sleep system in that they mediate arousal (Saper et al., 2010). The ORx projection pattern encompasses virtually all brain regions thought to be involved in sleep generation and regulation (Table 1; Ohno and Sakurai, 2008; Morin, 2013a). Phase of the oscillatory cellular SCN network (Hong et al., 2012; Freeman et al., 2013) is set by the prevailing photoperiod and the SCN output, in turn, appears to set the phase of ORx neurons (Deboer et al., 2004; Zhang et al., 2004; Martinez et al., 2009; Mahoney et al., 2013). A direct projection from the SCN to ORx neurons (Fig. 3B) has been reported, with many of the fibers originating in vasoactive intestinal polypeptide cells of the SCN (Abrahamson et al., 2001; Delaunay et al., 2009). The SCN efferents largely innervate medial and central ORx cells, with little input to those located laterally (Yoshida et al., 2006). SCN projections would be presumed to be GABAergic (Moore and Speh, 1993; Abrahamson and Moore, 2001; Morin and Blanchard, 2001) and largely inhibitory (but see Freeman et al., 2013 for discussion of alternate possibilities). It should also be noted that there is feedback from ORx neurons onto the SCN (Table 1). ORx release has direct and indirect inhibitory effects on SCN activity, and may be a key means by which sleep-/arousal-related stimuli modify circadian rhythm function (Belle et al., 2014).

**Table 1:** Retinorecipiency of selected brain regions and their innervation by orexin or MCH cells

Brain region	Retinal input^a^	Orexin terminals^b^	MCH terminals^c^
Sleep/circadian-related			
DM	–	++++	+++
DPGi	–	++	+++
DR	–	++++	+++
IGL	++++	++++	++^c^, –^e^
LC	–	+++++	++
LDTg	–	+	++
LDTgV	–	+	++
MnR	–	++++	+++
MPA	±	+++	++
MPO	–	+++	++
MTu	–	+++	++++
OPT	++++	–	++
PNo	–	+	++
PPTg	–	++	+++
SCN	+++++	+	++
SubCA	–	+	+++
VLPO	±	+	+
VTA	–	+	+++
			
Other retinorecipient			
CPT	+++	+++ ^d^	++
DLG	+++++	–	++
HDB	+	++	++++
InG	+++	+ ^d^	++
MeA	+	+++	+
MT	+++++	–^d^	++
Op	+++++	+^d^	++
PHb	+++	+^d^	++
PLi	++	++^d^	++
RCh	++	++++	++
sPA	++	+++	++
SuG	+++++	– ^d^	+++
Zo	+++++	– ^d^	+

Estimated density: extremely dense +++++; dense ++++; moderate +++; modest ++; sparse +; very sparse ±; none –.

a From Table 1 in Morin and Studholme (2014b)

b Hamster data from Table 1 in Mintz et al. (2001) and Table 1 in Horowitz et al. (2005) with reference to corresponding photomicrographs to resolve nomenclature ambiguities between Mintz et al. and Horowitz et al.

c Rat data from Table 2 in Bittencourt et al. (1992) or reference to corresponding photomicrographs to resolve nomenclature ambiguities.

d New data from direct examination of archived hamster tissue (Morin and Blanchard, unpublished observations).

e Hamster data from Vidal et al. (2005).

**Table 2:** Anatomical abbreviations

AH	anterior hypothalamic nucleus
CPT	commissural pretectal nucleus
DLG	dorsolateral geniculate nucleus
DM	dorsomedial hypothalamic nucleus
DPGi	dorsal paragigantocellular nucleus
DR	dorsal raphe nucleus
HDB	diagonal band nucleus, horizontal limb
IGL	intergeniculate leaflet
InG	intermediate gray layer, superior colliculus
LA	lateroanterior hypothalamic nucleus
LC	locus coeruleus
LDTg	lateral dorsal tegmental nucleus
LDTgV	lateral dorsal tegmental nucleus, ventral
LPBC	lateral parabrachial nucleus, central part
MeA	medial amygdala nucleus, anterior
MnR	median raphe nucleus
MPA	medial preoptic area
MPO	medial preoptic nucleus
MT	medial terminal nucleus
MTu	medial tuberal hypothalamic nucleus
Op	optic layer, superior colliculus
OPT	olivary pretectal nucleus
Pa	paraventricular hypothalamic nucleus
PHb	parahabenular area
PLi	posterior limitans nucleus
PNo	pontine reticular nucleus, oral part
PP	peripeduncular nucleus
PPTg	pedunculopontine tegmental nucleus
pSON	peri-supraoptic area
RCh	retrochiasmatic area
SCN	suprachiasmatic nucleus
SON	supraoptic nucleus
sPa	subparaventricular hypothalamic nucleus
SubCA	subcoerulean area
SuG	superficial gray layer, superior colliculus
VLG	ventrolateral geniculate nucleus
VLPO	ventrolateral preoptic nucleus
VTA	ventral tegmental area
ZI	zona incerta
Zo	zonal layer, superior colliculus

A multisynaptic, sleep regulatory route likely involving the VLPO and the ORx system has been postulated based on the results of axon-sparing neurotoxic lesion studies of the DM (Chou et al., 2003; Saper et al., 2010). This route consists of serial connections between the retina, SCN, sPa, DM, VLPO and from there, caudally to the perifornical lateral hypothalamic area (Fig. 3B). ORx neurons in this region receive input from VLPO neurons (Yoshida et al., 2006). This proposed avenue from the SCN may provide entrainment-related information to the sleep system (Saper et al., 2010). Inhibitory input arriving directly or indirectly from the GABAergic SCN neurons could make the ORx system responsible for time-of-day-dependent nREM sleep (Tsunematsu et al., 2011).

The brain stem sleep-regulatory nuclei known to receive projections from ORx neurons (Table 1) are reciprocally connected with the IGL (Horowitz et al., 2004; Morin and Blanchard, 2005). This creates a potential feedback loop whereby the sleep-related physiology may modify function of the SCN circadian clock, which is accessible via the geniculohypothalamic tract originating in the IGL (Deboer et al., 2003; Houben et al., 2009; van Oosterhout et al., 2012b; Morin, 2013a). Complicating matters further is the fact that the IGL itself is the recipient of ORx input (for discussion and references, see Morin, 2013a). This opens the possibility that the ORx system has both direct and indirect access to caudal sleep-regulatory nuclei, as suggested by its apparent influence on neurons in the dorsal raphe nucleus (Adidharma et al., 2012).

#### Melanin-Concentrating Hormone Pathways

The lateral hypothalamic region also contains a collection of neurons identifiable by their melanin-concentrating hormone (MCH) content. The distribution of these cells is broad and overlaps the distribution of ORx cells, but without neuromodulator colocalization (Broberger et al., 1998; Elias et al., 1998; Vidal et al., 2005). MCH and ORx neurons innervate many brain regions, most of which receive input from each cell type (Table 1; Bittencourt et al., 1992; Elias et al., 2008; Morin, 2013a). In addition, activity of MCH cells is anti-phase to ORx cell activity (Hassani et al., 2009) and the two neuron types appear to be reciprocally connected (Bayer et al., 2002).

Recent developments involving MCH have been the subject of significant discussion (Peyron et al., 2011; Fraigne and Peever, 2013; Jego and Adamantidis, 2013; Jones and Hassani, 2013; McGinty and Alam, 2013; Monti et al., 2013; Pelluru et al., 2013). MCH knockout mice are more active and sleep less than wild-type mice (Willie et al., 2008; Takase et al., 2014). Furthermore, optogenetic stimulation of MCH cells during the mouse nocturnal active phase increases sleep at that time (Konadhode et al., 2013; but see Tsunematsu et al., 2014 for a differing view). This result may be analogous to light-induced sleep. Collectively, the results suggest the presence of a sleep/arousal regulatory circuit, key elements of which are the MCH and ORx neurons.

#### MCH and ORx Interactions

One hypothesized version of MCH/ORx cell interaction (Pelluru et al., 2013) is that sleep-active preoptic neurons and lateral hypothalamic MCH cells enable sleep through inhibition of arousal-inducing neurons, including those of the ORx complex (Rao et al., 2008; Konadhode et al., 2013; Pelluru et al., 2013). A sleep episode may terminate because of MCH cell auto-inhibition and the wake interval may end as the ORx cells activate MCH neurons. Ultimately, the likelihood of sleep or wake may be related to the relative balance of ORx and MCH cell activity, although a direct sleep induction effect of MCH acting in the VLPO has been reported (Benedetto et al., 2013). It should also be noted that both ORx and MCH cells have been implicated in the regulation of metabolic and behavioral (feeding) responses to glucose signals. As with the conceptual framework underlying the relationship between ORx and MCH with respect to arousal and sleep, the two neuromodulators are thought to act in a complementary manner to promote and reduce energy expenditure (Barson et al., 2013; Pelluru et al., 2013). This information emphasizes the point that sleep regulation is not the exclusive function of ORx and MCH cells. Each neuromodulator influences multiple behavioral and physiological systems (Leonard and Kukkonen, 2014; Takase et al., 2014).

Presently, there is substantial imbalance in the research literature concerning the sleep-related activities of MCH and ORx cells. Investigation of MCH lags far behind ORx studies. For example, there is no information regarding whether or not MCH cells are innervated directly or indirectly by the retina. Nevertheless, because there appears to be a potent role for MCH in sleep/arousal regulation, especially in relationship to ORx cell function, the existing imbalance in favor of ORx is likely to be greatly eroded in the next few years.

#### Additional Anatomical Considerations

Several issues cloud the potential importance of the ORx or MCH systems with respect to light-alteration of sleep and arousal. The first is whether a neurohumoral signal elicited by retinohypothalamic input and emanating from the SCN might mediate light-induced sleep (cf., Silver et al., 1996). The notion of neurohumoral signaling has a certain appeal because it has been notoriously difficult to demonstrate, using knife-cut procedures, that particular SCN efferent projections regulate circadian rhythmicity (Nunez and Stephan, 1977; Inouye and Kawamura, 1979; Nunez and Casati, 1979; van den Pol and Powley, 1979; Brown and Nunez, 1986; Hakim et al., 1991; Harrington et al., 1993). In fact, SCN neural efferents may be more important for rhythmic endocrine function than governance of locomotor activity (Watts et al., 1989; Meyer-Bernstein et al., 1999).

A second issue concerns the presence of ORx in numerous retinal cell types, including ganglion cells that contain melanopsin photopigment and project to the SCN (Liu et al., 2011). This unexpected observation complicates interpretation of the functional neuroanatomical data, especially information obtained from studies in which systemically administered ORx agonists/antagonists are used as probes of the ORx system. Observed behavioral changes could be the result of a direct drug effect on either the retina or brain. Additionally, ORx released in the SCN from retinohypothalamic tract terminals could theoretically induce behavioral change that would not involve input from lateral hypothalamic ORx neurons.

Thirdly, numerous neuropeptides are present in cells of the lateral hypothalamic region (Gerashchenko and Shiromani, 2004; Horjales-Araujo et al., 2014). At least one of these, dynorphin, is almost completely colocalized with ORx (Chou et al., 2001; Bayer et al., 2002; but see Harthoorn et al., 2005). Dynorphin tends to be inhibitory, ORx excitatory, and the two neuropeptides are able to modulate each other’s actions (Li and van den Pol, 2006). Each induces changes in physiology and behavior that are not necessarily related to sleep and arousal (Li et al., 2014). Given the variety and, likely growing, number of neuropeptides in lateral hypothalamic cells and the possible interactions between them, it will be time consuming to sort out the importance of each relative to the various functions they collectively modulate.

## Practicalities for Sleep Research

### Photosomnolence Induction by Simple Stimuli

Mrosovsky typically employed 30 or 60 min light pulses to suppress nocturnal locomotion in mice and hamsters (Redlin and Mrosovsky, 1999b; Mrosovsky et al., 2000, 2001; Mrosovsky and Hattar, 2003), but millisecond stimuli are sufficient (van den Pol et al., 1998; Arvanitogiannis and Amir, 1999; Morin and Studholme, 2009; Morin et al., 2010; Morin and Studholme, 2011; Studholme et al., 2013). Numerous studies have shown the magnitude of locomotor suppression to be dependent on irradiance (Mrosovsky et al., 1999; Redlin and Mrosovsky, 1999b; Mrosovsky et al., 2000, 2001; Mrosovsky and Hattar, 2003; Thompson et al., 2008; Morin et al., 2010). The precise combination of irradiance, flash duration, number of flashes, and interflash interval that will elicit maximal suppression is not yet known, although four flashes (2 ms each) with a 16 s interflash interval yields a robust response (Morin and Studholme, 2009).

For the sake of methodological convenience, a 5 min bright (e.g., 100 µW/cm^2^) light pulse is most useful because of the typical ease with which it can be delivered and the fact that it elicits responses (Figs. 1, 2) equivalent to those produced by a series of millisecond flashes (Morin and Studholme, 2009, 2011). In most studies of photosomnolence, a brief light pulse or a series of flashes delivered over a brief interval is likely to be preferred to either type of photic stimulus delivered over a longer interval (30 − 60 min). The longer stimuli may unduly complicate interpretation of locomotor suppression or sleep induction experiments because of the additivity effects discussed above (Morin and Studholme, 2014a).

It is worth emphasizing that photosomnolence is a very practical research endpoint in studies of sleep induction and regulation in mice or hamsters, although a less robust form of it may also occur in rats (Borbély et al., 1975; Borbély, 1978). First, in mice and hamsters, induction of sleep is extremely simple, involving only brief light exposure. Second, the timing of the induced sleep is very much under the investigator’s control. Third, there is very little uncertainty regarding the timing of induced sleep because it occurs with a short latency after stimulus onset. Fourth, the induced sleep endures for a relatively fixed interval amenable to study across its duration. Fifth, arousal or recovery from sleep can easily be studied as part of the research protocol. Sixth, the pattern of photosomnolence is sufficiently stable and invariant within and across individuals that it is an excellent baseline of behavior against which the effects of drugs or other experimental manipulations can be compared. Finally, and especially with respect to point 6, any method that quantifies locomotion, including wheel-running measures, is likely to provide an index of sleep behavior that is adequate for many studies.

### Simplified Behavioral Sleep Assessment

Pack et al. (2007) devised a simple, effective, video-based method for translating the amount and timing of open-field locomotion into an index of sleep behavior. With it, animals are dichotomized as being asleep or awake according to easily defined behavioral criteria. Both Pack et al. (2007) and Fisher et al. (2012) note the method’s practicality for the rapid, simultaneous evaluation of sleep by numerous individuals. The results of video-based locomotor analysis have a very high correlation with EEG-determined sleep, although they are not useful for assessment of the REM component. Video-based sleep analysis has now been successfully employed by additional investigators (Morin and Studholme, 2011; McShane et al., 2012; Studholme et al., 2013). Other procedures devised to measure correlates of behavioral sleep, such as the respiration pattern, have also been established as useful alternatives to the EEG (Flores et al., 2007; Donohue et al., 2008; Zeng et al., 2012; Rowe et al., 2014). It is also important to note that none of these procedures require surgery.

**Figure 4.**
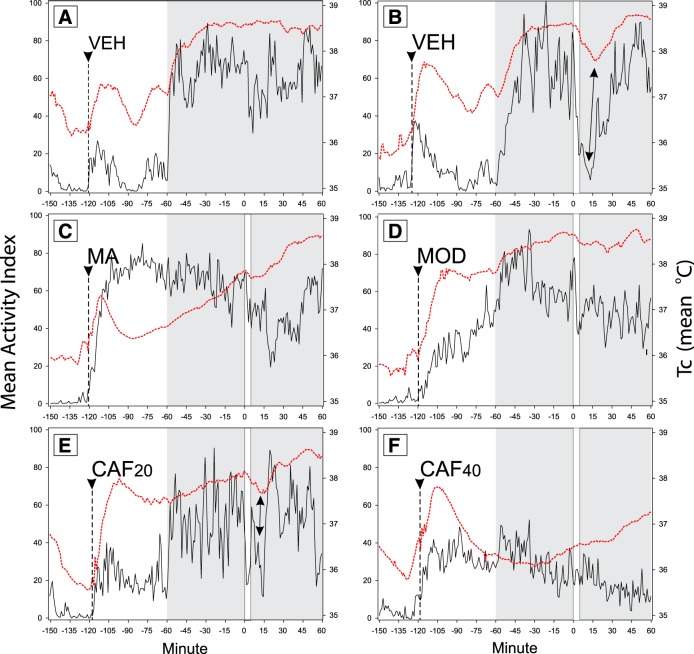
Mean simultaneously recorded activity indices (solid black lines; motion detected from video) and core body temperatures (broken red lines) for groups of mice injected with vehicle (VEH; ***A***, ***B***), methamphetamine (MA; ***C***), modafinil (MOD; ***D***), caffeine (CAF; ***E***, ***F***), 20 and 40 mg, respectively. The times of injection are indicated by the arrowhead and broken vertical line. The daylight period (white area) is to the left and dark period is to the right (shaded area). In ***A***, no light pulse was administered. In ***B−F***, a 5 min light pulse (vertical white area) began at time 0. The light-induced drop in mean locomotor activity and Tc is evident in ***B*** (double-headed arrow) and less so in ***E***. Modified from Figures 5-7 in Vivanco et al. (2013); see for details.

As emphasized by Pack et al. (2007) and Fisher et al. (2012), video-based analysis is particularly useful for testing sleep responses to pharmaceuticals. One example is a recent investigation that evaluated the effects of psychostimulant drugs on photosomnolence, as estimated from open-field locomotion (Vivanco et al., 2013). Each of three drugs administered to mice several hours prior to exposure to the photic test stimulus caused acute hyperactivity (Fig. 4). However, by the time of light exposure, the hyperactivity induced by modafinil or methamphetamine had ceased and the caffeine-induced hyperactivity of the third group had been replaced by significant hypoactivity. Most importantly, regardless of the level of activity at the time of light exposure, all three drugs prevented photosomnolence and the expected drop in Tc.

Such results redirect the research pathway back toward the ORx and MCH functions. The pharmacological activities of all three psychostimulant drugs are thought to involve the dopamine neurotransmitter system. MCH is known to act on this system as a partial dopamine D1 receptor agonist (Alberto et al., 2011). Stimulation of the D1 receptors suppresses sleep attacks and promotes wakefulness in mice lacking ORx (Burgess et al., 2010). Absence of the MCH1 receptor induces dopamine supersensitivity and locomotor hyperactivity (Smith et al., 2005, 2008). Dopamine receptors are also known to mediate light-induced locomotor suppression (Doi et al., 2006) and several dopamine-related circuits might be involved in the suppression response (for discussion, see Vivanco et al., 2013).

## Conclusion

Nocturnal light influences multiple systems governing physiology and behavior (Borbély and Huston, 1974; Morin, 2013b). These include sleep, locomotor suppression, body temperature, melatonin suppression, circadian rhythm phase control, adrenal corticoid production, and possibly others. In fact, the identical photic stimulus can have simultaneous effects on each system. Even in humans this may be the case, but with effects befitting the diurnality of the species. In particular, nocturnal millisecond light stimuli induce phase shifts in humans, while simultaneously reducing EEG-sleep and increasing alertness (Zeitzer et al., 2011). Because of the influence of light on multiple systems, it is unlikely to be a simple task to distinguish the extent of functional independence of each system or the interdependence of several systems. The most obvious fact about the various light-modulated systems is that they oscillate under the influence of the SCN circadian clock and, through that mechanism, they entrain to the daily photoperiod. The impact of each oscillatory system, such as the light-entrained rhythm of adrenal hormone secretion, on the function of the others (such as sleep), is more difficult to see at the present time.

A model has been proposed (Morin, 2013b) in which the SCN, site of the primary central circadian clock and chief component of the circadian rhythm system, is the first-order target of photic information affecting the response of each photically modulated system. According to the present understanding, there is little to no distinction between the first-order retinorecipient SCN cells and the cells comprising the circadian clock. However, light can induce locomotor suppression in mice lacking circadian clock function (Van der Horst et al., 1999; Mrosovsky, 2001). All other behavioral or physiological systems in which change is induced by nocturnal light are expected to be rhythmically modulated targets of the SCN circadian clock. To date, there has been no effort to relate light-induced changes in SCN cell activity to locomotor suppression and photosomnolence, although there is substantial information about SCN cell response to light (Meijer and Rietveld, 1989; Meijer and Schwartz, 2003; Drouyer et al., 2007; van Oosterhout et al., 2012a). In addition, no neurophysiological studies concerned with any light-regulated function of the SCN have considered the possibility that one or more of those functions (e.g., sleep induction) might have feedback effects on the cellular SCN activity being evaluated (Deboer et al., 2003).

With respect to the sleep generation and regulatory system, it is not clear how the light-induced change in Tc contributes to the likelihood of either locomotor suppression or sleep behavior. Despite the fact that there is substantial information supporting a role for thermoregulation in the control of normal sleep, the observed light-induced Tc and sleep responses could very well be parallel, but otherwise independent, event sequences. Considering that each system may have its own control circuitry, it is theoretically possible to separately destroy the pathways regulating each such system. This research tactic has been applied, without much success, in numerous studies designed to determine the general route of SCN-efferent projections providing temporal information to downstream systems generating the locomotion seen in circadian wheel-running rhythms. An advantage of the light-induction approach to sleep research is that the normal response is of the fairly clear all-or-none variety and one would expect fairly precise, properly placed, lesions to simply prevent photosomnolence. Although this approach has been applied to retinorecipient thalamus and midbrain, it has generally not been applied to hypothalamic structures other than the SCN.

Suprathreshold nocturnal photic stimulation results in behavioral state change with animals switching rapidly from aroused and highly active to inactive and asleep (Morin and Studholme, 2011). Although the exact latency to onset of the state change has not been determined, it is likely under a minute. At the level of the regulatory brain nuclei inducing sleep in response to photic input, function will change as if a switch has been thrown. The logic and literature supportive of “sleep state switching” has been discussed in detail by Saper and colleagues (2001, 2010). The premise is that collective action by one cell group inhibits a counteractive set of cells, turning them off in favor of the function of the inhibiting group. State change can occur when inhibition causes back and forth “flip-flop switching” between the current and prior states. Light-induced state change would appear to be efficiently and conveniently amenable to the study of sleep state switching precisely because light is a known trigger that is likely coordinating downstream cellular activity, while sharply reducing the variability in the sleep state transition time (for discussion, see Saper et al., 2010).

From a perspective of pure sleep research, further research is needed to clarify the nature of photosomnolence. For example, is the sleep response elicited by light “normal” in all its EEG characteristics? Is it additive to sleep homeostasis (e.g., provide a sleep accrual benefit to animals suffering from a sleep deficit)? If the answers to these questions are not in the affirmative, what is the significance of photosomnolence to sleep research and the understanding of sleep management?

In the future, a mixture of anatomical and functional investigations will likely provide a clear description of the photic input pathways to the sleep system. A research strategy designed to determine the neural pathways by which photic input reaches and activates the sleep system would seem not only practical, but applicable to the other behavioral and physiological systems modulated by nocturnal light. It seems appropriate to end by reiterating the final point in Borbély’s review: “Light, a natural environmental variable, constitutes a promising tool for experimental investigation of sleep and activity, and their rhythmic variations” (Borbély, 1978, p. 26).

## Synthesis

The decision was a result of the Reviewing Editor Cheryl Sisk and the peer reviewers coming together and discussing their recommendations until a consensus was reached. A fact-based synthesis statement explaining their decision and outlining what is needed to prepare a revision is listed below. The following reviewer agreed to reveal his identity: Bruce O’Hara

Two experts in the field and I have reviewed your manuscript and we agree that the review is timely and of interest to the readership of eNeuro. The two reviewers have each made several suggestions for improving the review, and I ask that you please respond to their suggestions, either by revising the manuscript accordingly, or state why those revisions should not or cannot be made.

### Reviewer 1

This is an extremely well written review paper, it is very thorough, timely and informative. It is also written in a very accessible style so as to not be too simplistic for experts, but not too technical for novices.

1. the title seems a bit general given the fact that the review focuses on light induced sleep in nocturnal rodents.
2. I wonder if the authors could compare the sleep bouts induced by photic stimuli at night with those occurred naturally during the day or night, and discuss the extent to which they are similar or different.
3. the three lines in Fig 2 are hard to tell apart. colored lines would be helpful.
4. the work of light induced behavioral changes in diurnal rodents are cited. elaborate those work in a separate section may help to broaden/extend the scope of the review.

### Reviewer 2

This article discusses the important and under-appreciated phenomena of photosomnolence and more generally the many effects light has on the brain and body. This includes the important interactions of light, sleep, temperature (and the complex interplay of core body temperature, hypothalamic brain temp, peripheral temp etc.) and provides a critical review of what is known, and more importantly what is not known in these areas. This is an important synthesis of previous research and new ideas. Many of the proposed mechanisms are speculative, but are testable, which provides much of the value of this manuscript.

For example, I am not convinced of a central role for the SCN in photosomnolence, or of the orexin system for that matter, but more careful studies of SCN and orexin lesions can test these ideas more directly.

minor edits:

line 69: typo or missing words in " as occurs repeatedly animals" but more importantly is "triggered" overstating the data from the that paper, when really more of increase over basline in REM sleep.

line 311: maybe include this smaller average change in temp to contrast with the large 1.7 degree change at CT15.

line 370-374: are the lack of direct brainstem connections referring to a lack of connections to the LC, raphe nuclei, etc. If so, might this just argue that these regions are not really involved (yes they correlate with arousal state changes and CAN alter arousal states, but they may in general be secondary effects).
